# Real-Time Curvature Defect Detection on Outer Surfaces Using Best-Fit Polynomial Interpolation

**DOI:** 10.3390/s121114774

**Published:** 2012-11-02

**Authors:** Ehsan Golkar, Anton Satria Prabuwono, Ahmed Patel

**Affiliations:** 1 Center for Artificial Intelligence Technology (CAIT), Faculty of Information Science and Technology, Universiti Kebangsaan Malaysia (UKM), 43600 UKM Bangi, Selangor, Malaysia; 2 School of Computing and Information Systems, Faculty of Science, Engineering and Computing, Kingston University, Kingston upon Thames KT1 2EE, UK; E-Mail: whinchat2010@gmail.com

**Keywords:** visual inspection system, flatness, waviness, surface defect detection system, curvature measurement

## Abstract

This paper presents a novel, real-time defect detection system, based on a best-fit polynomial interpolation, that inspects the conditions of outer surfaces. The defect detection system is an enhanced feature extraction method that employs this technique to inspect the flatness, waviness, blob, and curvature faults of these surfaces. The proposed method has been performed, tested, and validated on numerous pipes and ceramic tiles. The results illustrate that the physical defects such as abnormal, popped-up blobs are recognized completely, and that flames, waviness, and curvature faults are detected simultaneously.

## 1. Introduction

Real-time visual inspection systems have improved over the last few years, ensuring a high standard of product quality in the mass production systems of industry. This is compounded by the fact that manufacturing industries are strongly motivated to use high-precision fault and defect detection systems with minimum development, low installation and maintenance costs, and a reduction in avoidable, time-consuming effort. Due to these requirements, applying a powerful inspection system with the ability to inspect and detect all kinds of faults is of significant interest. To create a complete, real-time defect detection system, it is essential to know all the faults that result in defects. In terms of product quality control, surface geometry and physical properties are often classified into four groups:

- Defects, such as: welds, spots, blobs, cracks, and scratches in the ceramic-tile industries;
- Roughness, such as residual polishing-marks in the car industries;
- Waviness, although not as common as roughness, has a measurement. Measuring waviness in a bearing ball is one example.
- Flatness, roundness, or curvature, which is an error on the shape of products. Flatness faults of stones or ceramic tiles are typical examples.

These defects are classified as irregular patterns on these surfaces upon inspection. This paper gives a background to the problem through summarizing related works and problem statement in the subsections below. Section 2 presents, in detail, the novel methodology adopted to resolve the problem. Section 3 renders the validation and the measurement accuracy of the proposed method for various lines and ellipses. Section 4 explicates the results of the proposed method, which was tested in real-time on ceramic tiles and pipes. Finally, the paper is wrapped up by a comparison, discussion and an overall conclusion.

### 1.1. Background

Rosati *et al.* employed a non-flat mirror to illuminate and inspect highly reflective, curved surfaces. Using a mirror causes reflected noises to decrease, and a camera captures the surface indirectly, with less noise [1]. Jia *et al.* proposed a real-time inspection system for steel surfaces with complicated defect patterns. The strategy used a supported vector machine to learn pattern defects [2]. In another method, the combination of machine-vision and laser beam was applied to measure the flatness and straightness of planes. In this study, a beam laser projects onto the steel surface, and a camera with 45° angle, is located on the laser plane. The captured image is then processed by a featured extraction method based on the Legendre polynomial fit [3]. Sun *et al.* proposed an automated vision system, based on fuzzy pattern recognition, to inspect steel tubes [4]. Abdelkhalek *et al.* proposed a combination of elastic deflection, the thermal crown and the roll grinding crown to inspect flatness and waviness defection in thin plates. The proposed method is appropriate for center waves and edges waves produced in sheet metal during rolling [5]. In a following study, research reviewed surface inspection techniques that used light scattering and active vision. Afterwards, an innovated, integrative method for multi-parameter area was presented [6]. Jai *et al.* introduced a model by utilizing a fuzzy neural model for flatness pattern recognition with Legendre orthodoxy polynomial as a basic pattern. The result was practical effective to find flatness pattern defects, with strong self-adaptability and anti-interference ability to dispose data while recognizing flatness [7]. Usamentiaga *et al.* presented a simple and maintainable method to investigate steel manufactures. By using a laser ranging technique, based on shape-line meter approaches, a configurable real-time method was proposed [8]. Chen *et al.* presented the automated defect detection system applied to bearing cylindrical surfaces, in which the performance of the system was related to pre-defined functions [9]. Abraham *et al.* proposed a machine, vision-based system to detect highly reflective chrome-coated rings [10]. Zapata *et al.* proposed an automatic system to detect, recognize, and classify welding defects in radiographic image. Artificial Neural Network (ANN) and Adaptive-Network-based fuzzy inference system is employed to classify the defects [11]. Li and Ren provided a real-time visual inspection system for discrete surface defects of rail heads. Local normalization method is employed to enhance the contrast of the rail image. The defect localization uses projection profile (DLBP) [12].

### 1.2. Problem Statement

A typical visual inspection system contains a camera, image-processing software, and a manufacturing process control system [13]. Other requirements such as sensors or laser beam lights are applied besides the cameras to complete the investigation system. The major problem of existing methods is that they are specialized for a unique kind of defect; sometimes related works have introduced a general method, but that the general method is not accurate [14]. A lack of a visual inspection system to investigate the flatness of outer surfaces without using extra equipment, such as a laser beam or an X-ray, is another major problem. This paper deals with these problems and attempts to present a generic method to inspect curvature and blob defects in products such as cylindrical surfaces or on the edges of items.

## 2. Curvature Measuring Using Best-Fit Polynomial Interpolation

**Figure 1.**
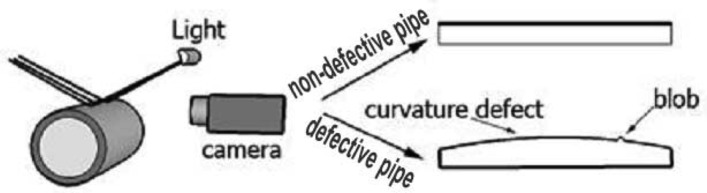
Camera captures image from side of pipe. The first image shows a non-defective pipe, and the second image depicts a pipe with curvature and blob defects.

Previous works concentrated their efforts on capturing and analyzing the outer surfaces of spheres and cylindrical objects by locating the camera above these objects [1]. In our proposed method, the camera inspects the outer surface of a cylinder from the side, as illustrated in Figure 1. In this state, the camera captures the outer surface of the cylinder, and from a mathematical point of view, if the curvature is zero, then the item is not defective. On the other hand, a defective item will have higher curvature values.

Any gain, however, should be seen in a wider context. If the camera captures outer surface from the side, it will not be able to see scratches, holes and small spots on the outer edges. Therefore, the presented method is proper to assess outer surface defects that change the curvature of surfaces and visible to the camera; especially for flatness defects, waviness blobs and welds on the edges of outer surfaces.

**Figure 2.**
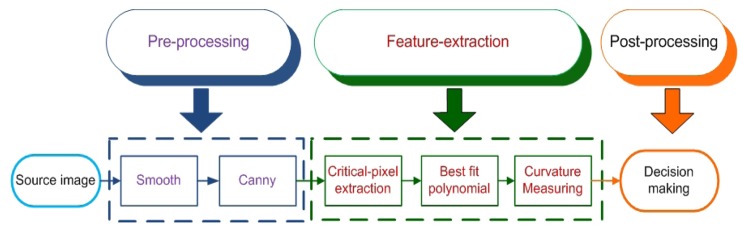
Overview diagram of proposed method.

In analogue view, all edges with different slopes seem smooth. Even all curves are easy to recognize, but in the digital view of an image, lines with different slopes have various breaks. Edges of images have various line segments, such as a curve, a circle, and a straight line [15]. Therefore, it is necessary to omit extra pixels that make digital lines abnormal. The whole process of the proposed method is depicted in Figure 2, which divides the presented methodology into pre-processing, feature-extraction, and post-processing phases.

In the pre-processing step, the image should be smoothed to decrease noise. A simple smoothing filter, such as a mean or median filter, is applied. Thereafter, the edges of the image are extracted using a canny edge detector [16], which uses a multi-stage algorithm to detect a wide range of edges in the image. It is noteworthy that in this paper we want to measure the curvature of outer surface; therefore, after using canny edge detection, extra edge images should be omitted. For this purpose, only the outer edge of images will be kept and all extra edges inside the outer edge will not be assessed. Then, from the top of the image a line that shows the outer surface of the object is retained. Finally, the edges of the image are sent to the feature extraction process, which contains the following three steps:

### 2.1. Critical-Pixel Extraction

The edges of the image source from the pre-processing step are sent to a feature extraction. These edges are called a digital image, but the source image does not match exactly because of resolution limitations. Therefore, this step matches a digital image to a source image and helps provide more accurate results. The aim of this step is to find the closest pixels in a digital image that are near to source image. In the proposed method, the coordinates of edges in an image are saved in *x* and *y* variables. Thereafter, the first and last pixels that are in the straight edge are extracted, and saved coordinates are compared to each other. The results of this comparison are divided to three categories:

- **Horizontal pixels**: The first and the last pixel on an edge that has the same *x* coordinate and a different *y* coordinate.
- **Vertical pixels**: The first and the last pixels on an edge that have the same *y* coordinate and a different *x* coordinate.
- **Single pixels**: Isolated pixels without neighboring pixels with the same *x* and *y* coordinates.

**Figure 3.**
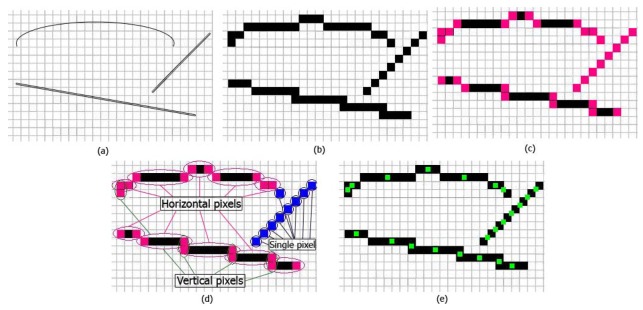
Example of finding critical-pixels. The grid shows pixels in a digital image: (**a**) image before using canny edge detector; (**b)** segmented edge image in digital form; (**c**) pink pixels are isolating pixels, or the first and the last pixels of straight lines; (**d**) horizontal pixels are in a horizontal line and vertical pixels are in a vertical line, and single pixels do not have any neighbor with the same x and y coordinates; (**e**) Green dots represent the average coordinate of each pair of horizontal, vertical, and single pixels.

The average of each category illustrates the pixels the closest to the source image. For instance, Figure 3 presents critical pixel-extraction stages, step-by-step. As shown in Figure 3(a,b), the source image and digital image (extracted by the canny edge detector) are shown, respectively, in the grid. The source image is closer to reality as exact matches rather than the digital image, because the digital image is limited in the number of pixels representing the image due to low resolution. Therefore, every pixel of an edge appears in either direct, straight edges or in single dots, which are depicted in Figure 3(b). As mentioned previously, by finding the first and last pixels of direct straight edges as shown with pink pixels in Figure 3(c) there is three categories [Figure 3(d)]. In the final step, the average coordinates of these three categories of pixels are critical-pixels, as shown in green in Figure 3(e).

### 2.2. Polynomial Function Extraction

From the previous step, the critical-pixels are collected as data points. These data points are used in polynomial interpolation. In mathematics, a polynomial is an expression of finite length, constructed from variables (also known as in determinates) and constants, using only the operations of addition, subtraction, multiplication, and non-negative integer exponents. Polynomial interpolation is a generalization of linear interpolation, and linear interpolation is a method of curve-fitting using linear polynomials. As mentioned, all extra edges of canny edge detection can be omitted, and only the edge of outer surface remains for processing.

Assume we have n critical-pixels after applying canny edge detector on the outer surface; therefore we have *n* data points and there is exactly one polynomial of degree at most *n* − *1* going through all data points [17,19]. A function that shows a set of data points is called interpolation if it crosses through those points. The function *y* = *f(x)* interpolates the data points *(x*_1_*, y*_1_*)* … *(x_n_, y_n_)*, if *f(x_i_)* = *y_i_* for all of 1< *i* < *n*. One famous method to extract the interpolation function is Newton' s divided difference formula. In this paper, all *n* critical-pixels will be given *(x_1_, f(x_1_))* … *(x_n_, f(x_n_))* , the divided difference are defined as follows *k_th_* divided difference is *f[x_k_* − *x_k_*_+1_*]*. In other words, *k* presents a degree of polynomial function, approximately:

(1) $$f[x_{k}]=f(x_{k}),f[x_{k}‐x_{k+1}]=\frac{f(x_{k+1})-f(x_{k})}{x_{k+1}-x_{k}}$$

(2) $$f(x)=f(x_{1})+\ldots +f[x_{1}\ldots x_{n}](x‐x_{1})\ldots (x‐x_{n‐1})$$

There is only one interpolating polynomial of degree, *n* − *1* or less for *n* points [14]. It is believed that when a number of data points are too large, the calculation of a polynomial function is difficult or impossible. Therefore, by representing the approximating polynomial function with lower accuracy, it is possible to extract the polynomial function. However, the approximated polynomial function or polynomial fitting function is not accurate, but the interpolation-error is calculable. Assume that *p*(*x*) is the (degree *n* − 1 or less) interpolating polynomial fitting *n* points; then, the interpolation-error is the difference between *f*(*x*) and *p*(*x*) [19], such that:

(3) $$f(x)-p(x)=\frac{\left(x-x_{1})(x-x_{2})\ldots (x-x_{n}\right)}{n!f^{\left(n\right)}(c)}$$

where *c* lays between the smallest and the largest number *x*, *x*_1_ … *x_n_*, and *f^(n)^* is an order derivative function in *c* point [20]. Therefore, with interpolation-error, we can define the accepted accuracy.

### 2.3. Curvature Measuring

Curvature refers to any number of loosely related concepts in different areas of geometry. Intuitively, curvature is the amount by which a geometric object deviates from being flat, or straight in the case of a line. The curvature of a circle is equal to the reciprocal of the radius:

(4) k=1/r

**Figure 4.**
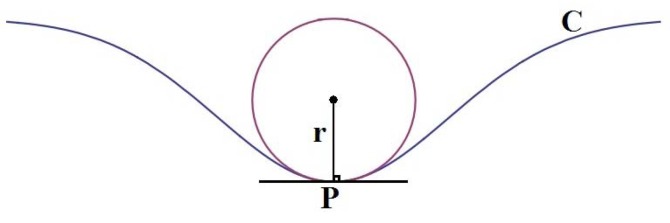
The curvature of point *p* on Curve *C*, is a circle with *r* radius.

On the other hand, the curvature of circles has an inverse proportion with the radius; increasing radius decreases curvature. Therefore, a circle with a large radius has a small curvature, and *vice versa*. Assume that *f(x)* is derived from Section 2.2, and all of critical pixels of outer surfaces should be assessed for curvature. Therefore, we have point *p* in a plane, and we want to calculate the curvature of that point. The presumptive circle that approximates the curve that *p* is the curvature of the plane on point *p* (see Figure 4) [18].

The curvature of point *p* with a polynomial function can be expressed as follows:

(5) $$k=\frac{d^{2}y/d^{2}x}{{\left[1+{\left(d_{y}/d_{x}\right)}^{2}\right]}^{3/2}}$$

where *dy/dx*, the first derivation and *d*^2^*y/d*^2^*x* is the second derivation of the polynomial function, and *k* presents the curvature values of the polynomial function. If curvatures of all n critical-pixels are obtained by Equation (5) by calculating one by one, *n* curvature values (*k_n_*) illustrate the curvature of outer surface in critical-pixels. Therefore, it is able to inspect the valid or invalid curvature from the outer surface.

## 3. Validation of Proposed Curvature Measurement

**Figure 5.**
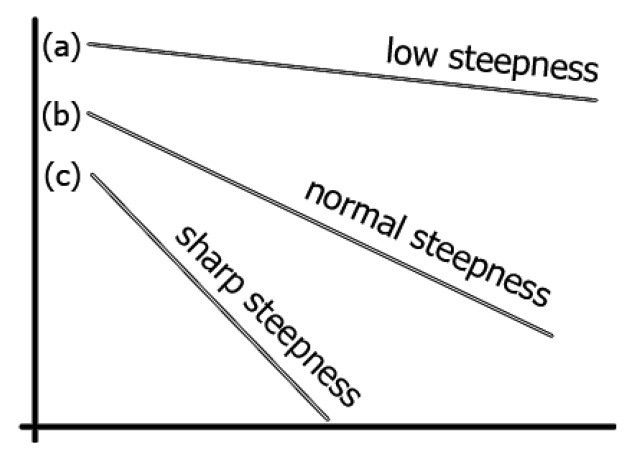
Three lines with different slopes showing different gradients: (**a**) Low steepness; (**b**) Normal steepness; and (**c**) Sharp steepness.

To validate the accuracy of the proposed method, three lines with different slopes are selected, as shown in Figure 5. Figure 5 Line (a) has a less or lower steepness when measured in angles as a gradient than Figure 5 Line (b), which is less steep than Figure 5 Line (c), which has a sharper gradient. The curvature of each slope should be zero, and the result of the proposed method verifies this fact.

**Figure 6.**
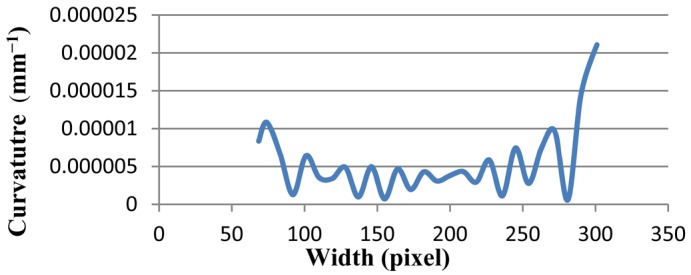
The curvature diagram of Line (a) in Figure 5 has less steepness.

The line graph of Line (a) is depicted in Figure 6. The curvature value stands at 8.0E-06 at pixel 70, at first. Thereafter, it fluctuates dramatically, around 5.0E-06 by pixel 280, and then hits a peak to about 2.0E-05 at pixel 310. Overall, the line-graph hovers around 5.0E-06, which is very close to zero.

**Figure 7.**
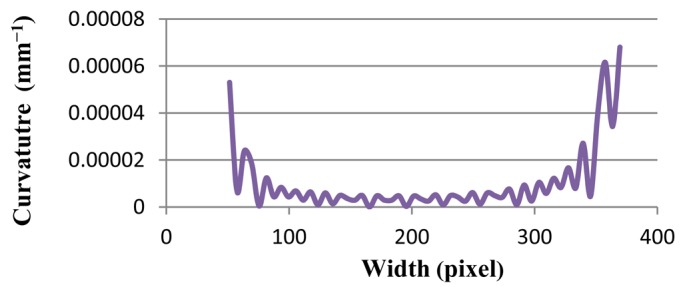
The curvature graph of Line (b) in Figure 5 has normal steepness.

**Figure 8.**
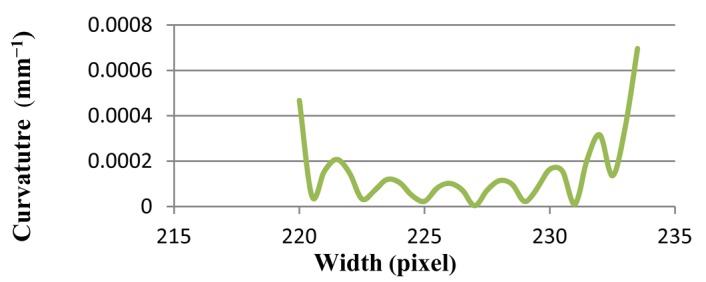
The curvature diagram of Line (c) in Figure 5 has sharp steepness.

The results of Line (b) and Line (c) are shown in Figures 7 and 8, respectively. Curvature value of Line (b) stands at 5.0E-05 at pixel 50. It then decreases sharply, near to zero, and continues its fluctuation around zero by pixel 350. Finally, the curvature mounts to less than 7.0E-05. At the same time, line graph of Line (c) hovers around zero, but it hits a peak at 7.0E-04 at pixel 230. All of these results show that the proposed method is stable with various gradients.

**Figure 9.**
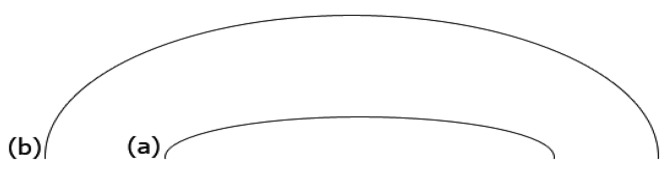
Two horizontal ellipses with different heights. Ellipse (**a**) is a narrow ellipse with large curvature in sides; and (**b**) has less curvature.

**Figure 10.**
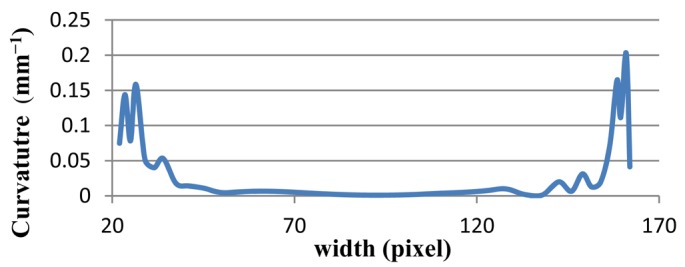
The line graph shows the curvature values of Ellipse (a).

**Figure 11.**
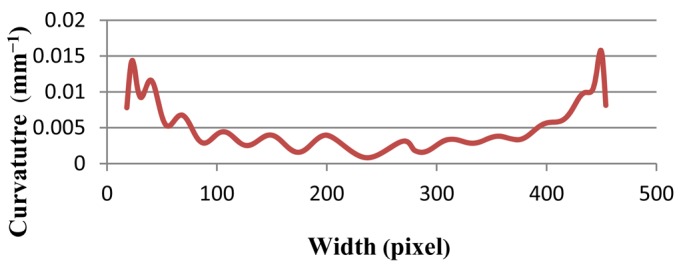
The line graph shows the curvature values of Ellipse (b).

To complete the validation, two ellipses are selected. The ellipse is selected because the curvature of two corners of the ellipse is more than in other parts. In Figure 9, the two horizontal ellipses are drawn. The curvature of the left and right parts of both should be larger than that of other points. In addition, the right and left sides of Figure 9(a) have more curvature than the ellipse in Figure 9(b). The proposed method is tested on both ellipses, and results have been presented in Figures 10 and 11 of Ellipse (a) and Ellipse (b), respectively. At the onset in Figure 10, the curvature stands at approximately 0.1. Afterwards, it fluctuates regularly, around 0.1 in 20 pixels. Thereafter, it plummets to zero at pixel 50, which is followed by a plateau at 100 pixels. Finally, the curvature mounts significantly to 0.1, and experiences fluctuation around 0.15 at pixel 170. In contrast, the Figure 11, illustrates curvature values of Ellipse (b). The curvature values stand around 0.01. The curvature then decreases to almost zero at pixel 250. Thereafter, the curvature increases to 0.01 at pixel 450. On the whole, both line graphs have large curvature values at the beginning and ending, while other points have smaller values.

Table 1 compares the results of the proposed method that contains maximum, minimum, average, and standard deviation of curvature values. Standard deviation shows how much variation or dispersion there is from the average (mean, or expected value). A low standard deviation indicates that the data points tend to be very close to the mean. The standard deviations of all lines [Line (a), Line (b), and Line (c) in Figure 5] and Ellipses (Figure 9) are very small.

## 4. Real-Time Testing of Proposed Curvature Measurement Method

In order to validate the inspection of the proposed system, an automated visual inspection system is proposed. The system is developed by a CCD camera with a maximum frame rate of 30 Hz. Furthermore, the distance between the camera lens and objects on the conveyor belt is 18 cm approximately. The light resource is a fluorescent lamp which illuminates a conveyor belt with an approximate 565 lx intensity from the top of the conveyor. As a result, most of reflection returns to the top of the inspected item and little light is visible to the camera. Therefore, the camera will be able to capture from high reflective surface. Moreover, a portable laptop with 2.2 GHz processor and 2 GB RAM is used to process the captured images.

### 4.1. Ceramic Tile Prototype Layout

**Figure 12.**
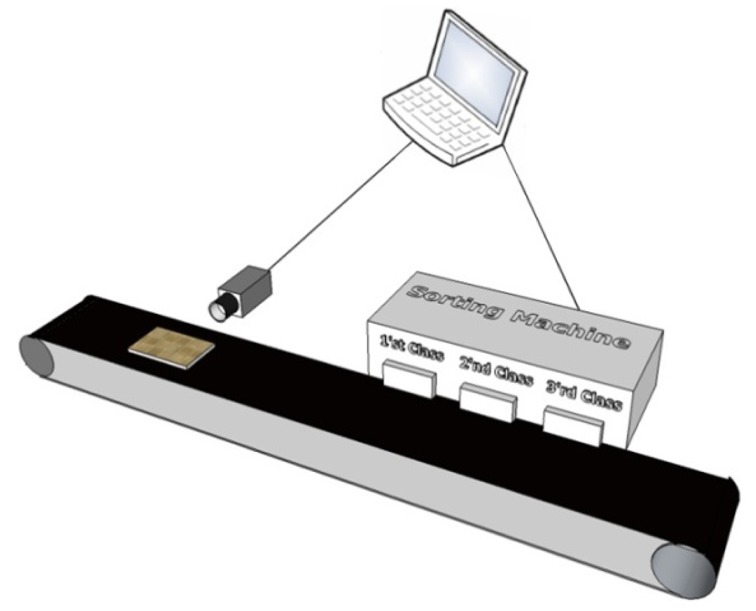
Layout of ceramic tile inspection system to capture the surface defects from the side.

**Figure 13.**
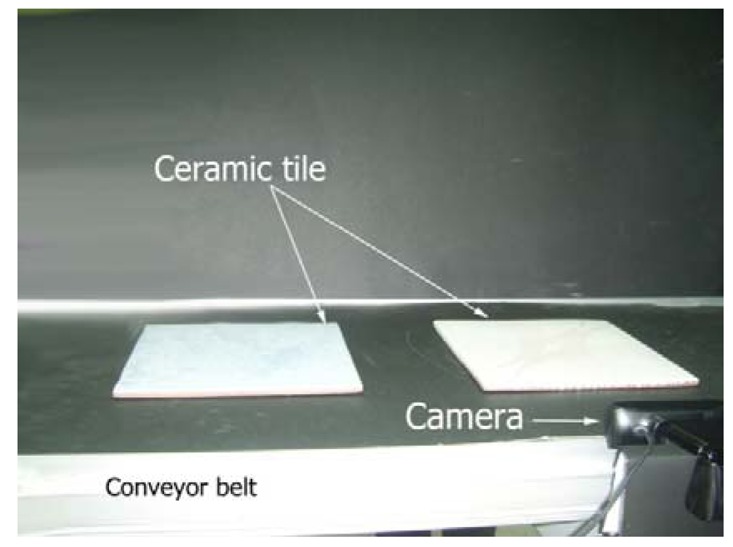
Close up of the ceramic tile inspection system layout.

The layout of a real-time system that investigates the ceramic tile edges is shown Figure 12. The camera captures the edges of a ceramic tile while the conveyor belt moves at a speed of 2.3 mm/s. The image with a resolution of 864 × 480 pixels is captured in a simple, white light that is illumined from the top (Figure 13).

### 4.2. Experimental Results of Ceramic Tile Testing

**Figure 14.**
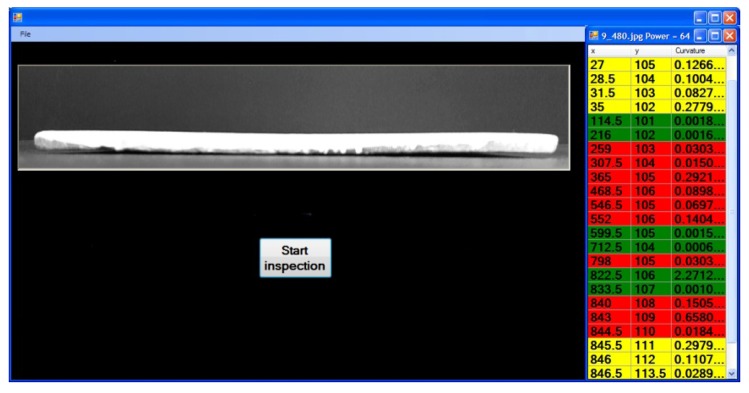
Ten defected points detected on the surface of a ceramic tile.

The software was developed in Visual Studio with OpenCV (image processing library) and ALGIB (mathematics library). The interface application is depicted in Figure 14. The software illustrates the online image captured on the left, while the right shows the curvature values of critical-pixels. The results indicate that the surface of the ceramic tile is not flat in ten crises points, which are shown in red. About 200 ceramic tiles were tested to validate the proposed method in a real-time system. These tiles had various defects, such as blobs, welds, cracks, and flatness. The system was able to detect all the defects on the edges of the ceramic tiles. The maximum valid value of a good ceramic tile was categorized by +1.0 mm^−1^ curvature. To increase the precision of the result, the setting of the camera was changed to a higher resolution (1,280 × 720 pixels), and the accuracy was increased to approximate +0.33 mm^−1^ curvature. In addition, the speed of using proposed algorithm in our system in an image with a resolution of 864 × 480 pixels is 0.31 s that increased to 0.43 s in images with 1,280 × 720 pixels. This speed indicates our system is quite rapid for real time inspection in ceramic tiles factory. This system can measure the flatness and waviness of ceramic-tiles as well; however, other defects, such as blobs and welds, are found more easily than curvature defects.

**Figure 15.**
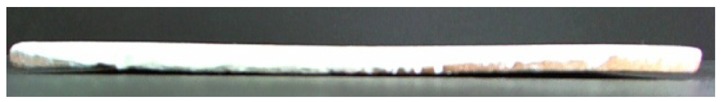
Source image of flatness defected tile.

**Figure 16.**
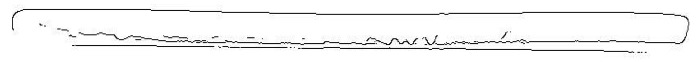
Edges of source image after applying canny edge detector on source image.

**Figure 17.**
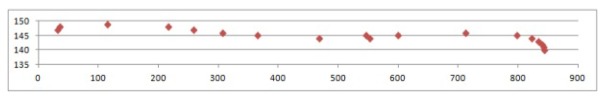
Critical-pixels of ceramic tile that has flatness defect.

**Figure 18.**
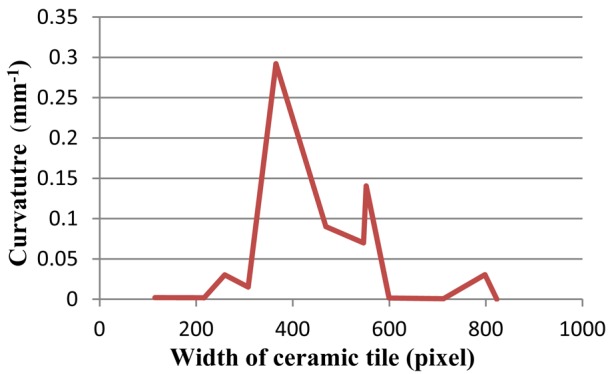
The curvature diagram of flatness defected tile.

Figures 15–17 depict a tile with a flatness defect, edges of ceramic tile, and critical-pixels, respectively. The curvature of critical-pixels is shown in Figure 18. In pixel 100, the curvature stands at zero. Thereafter, it hovers around zero at first, and then continues its upward trend to 0.3 in pixel 400. Afterwards, it experiences a plummet to less than 0.1 in pixel 500. The curvature then soars to 0.15, which bottoms-out to zero by pixel 600. Finally, it stabilizes at approximately zero. Overall, it is clearly seen that the tile has a flatness defect between pixel 300 and 600.

**Figure 19.**
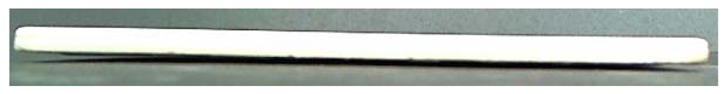
Source image of a non-defective ceramic tile.

**Figure 20.**
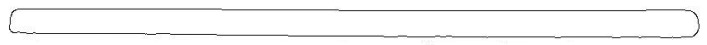
Edges of ceramic tile after applying canny edge detector on source image.

**Figure 21.**
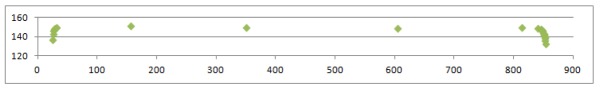
Critical-pixels of a non-defective ceramic tile.

**Figure 22.**
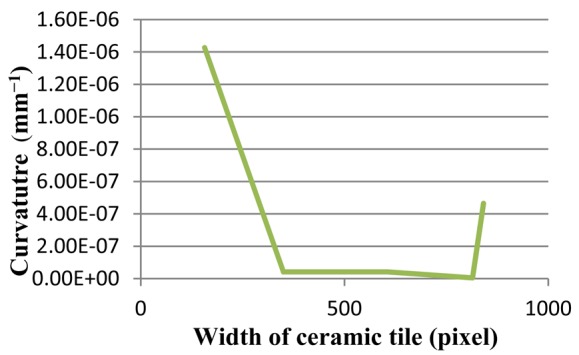
Curvature diagram of a non-defective ceramic tile.

In contrast, Figure 19 illustrates a non-defective ceramic tile. The proposed method has been tested on this tile, and the results of the curvatures are shown. Figure 20 depicts the image after the image pre-processing step. Afterwards, critical-pixels of Figure 20 are illustrated in Figure 21. Thereafter, the curvatures of critical-pixels are presented in Figure 22.

It is inferred that the curvatures of critical-pixels on the surface of the tile are less than 1.50E-06, which means it is a non-defective tile. This result depicts that the proposed method is able to classify non-defective tiles from flatness-defective tiles.

### 4.3. Pipe Prototype Layout

**Figure 23.**
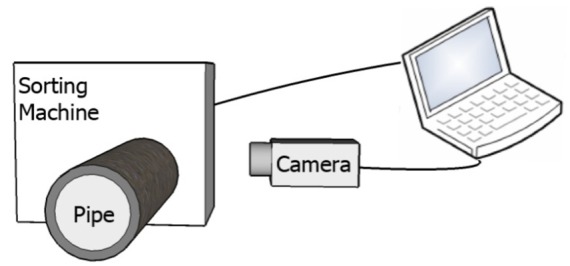
Layout of pipe inspection system with respective placements of a camera, pipe, and computer.

The proposed method was also tested on the outer surface of a steel pipe, which has high light reflection in a captured image. The layout of the steel pipe inspection system is illustrated in Figure 23. In this system, the camera captures the image of the steel pipe from the side. Next, the image, with a resolution of 864 × 480 pixels, is sent to the computer. After analysis, the rejected or accepted order is sent to the sorting machine by the computer.

### 4.4. Experimental Result of Reflective Pipe Testing

**Figure 24.**
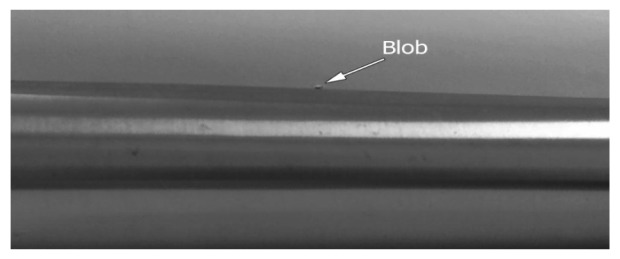
Real image of a reflective pipe showing a small blob on the surface.

**Figure 25.**
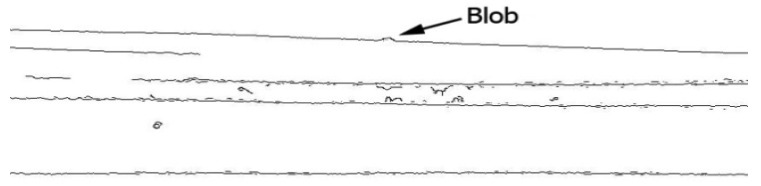
The small blob is shown on the surface of the pipe after applying the canny edge detector.

**Figure 26.**
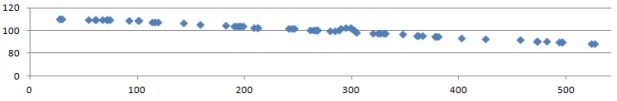
Critical-pixels of pipe with blob defect on the outer surface.

**Figure 27.**
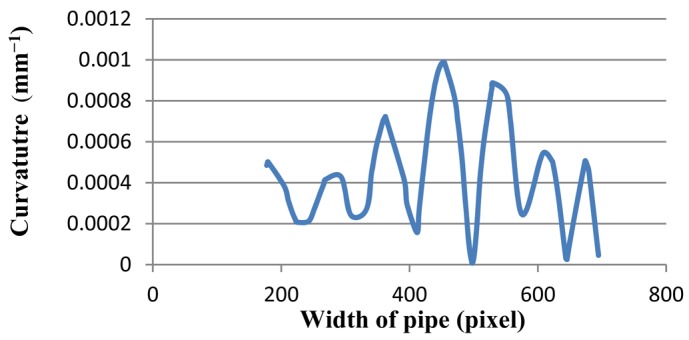
The curvature diagram of a blob defective pipe.

Since color, shadow, and reflection of light influence the image, the proposed system has been tested for several steel and coloured pipes. All experimental results indicate that the proposed method is robust, even for reflective and colorful, multi-curvature surfaces. Moreover, the results illustrate that not only is the proposed method able to assess blob defects in pipes, but it is also reliable to inspect waviness and flatness. Figures 24 and 25 depict the edge of a steel pipe with a small blob on the surface. Figure 26 shows critical-pixels of pipe with blob defect on the outer surface. Finally, in Figure 27, the curvature values of a blob defective pipe are shown. The line-graph fluctuates dramatically around 0.0004, which means that the surface is nearly flat. However, when the curvature reaches around 0.001 at pixel 450, it means that there is a defect in this part of the pipe. More experiments in various defects are shown in Appendix.

## 5. Comparison and Discussion

The proposed method has been tested on various ceramic tiles and pipes. The results illustrate that this method has the capability of sufficiently detecting the surface defects. Furthermore, it is possible to define the range of valid curvature defects. For example, if it is essential to inspect the outer surface meticulously, the interpolation-error mentioned before, *f(x)* − *p(x)*, should be very small. On the other hand, if it is needed to inspect the surface with less accuracy, the accepted curvature value should be greater. In our experiment, ceramic tiles should be inspected for flatness defects. We then reduced the accepted interpolation-error by 0.001, which is proper for the detection of flatness defects. Moreover, the curvature values of a ceramic tile surface, which were achieved from the proposed method for flatness controlling, should be very near to zero, and we selected 1.00E-05 for the accepted curvature value, ensuring that ceramic tiles are inspected accurately. However, in the other experiment, pipes were inspected for blobs. The accuracy of curvature measurements for pipes should be less than ceramic tiles, because blobs have more curvature values rather than flatness faults. Thus, the threshold errors for pipes are selected as 0.01 and 1.00E-03 for interpolation-error and accepted curvature, respectively. All results show that the lower the interpolation-error, the more accurate the curvature measurement. In addition, images with higher resolution were inspected more accurately than images with lower resolution. In the final analysis it is recommended that to inspect surfaces with high precision, the system should install high-resolution cameras and use low interpolation-error to achieve more accurate results. Compare to the other methods such as [1,2,4,5] that inspect outer surface for blob, weld and spot, our method is only able to detect blobs and welds approximately as large as 0.3 mm. Moreover, methods in [3,5–7] are specified to find flatness and waviness defect of outer surface, our method is accurate to find flatness with 0.33 mm^−1^ deviation. The difference between the proposed method and related work is that our method can find blob, weld, flatness and waviness defects simultaneously which is not seen in related work. However, the precision of detecting blobs, welds and spots is not as good as that of related work, but the accuracy of finding flatness defect on wavy edge is improved compared to related works.

## 6. Conclusions

A real-time visual inspection method for curvature and wavy edge defect detection of outer surfaces was proposed. The approach described an enhanced feature extraction method based on polynomial interpolation to detect flatness and waviness defects on outer surfaces. The proposed method introduces a novel method which finds critical pixels of an image; then, by utilizing polynomial interpolation, the curvatures of outer surfaces are assessed. It is noteworthy in the proposed method; the camera inspects the outer surface of items from the side. Therefore, less surface reflection is captured by the camera, and it is thus suitable for capturing images from reflective surfaces, although in this situation, small holes, scratches and cracks are hidden from the camera. Results of the proposed method, which has been tested on different graphical lines, ellipses, real tiles and pipes, indicate that it is able to measure curvature defects and wavy edges for various surfaces, such as colorful or reflective surfaces. Moreover, the outcome of testing 200 colorful and reflective ceramic tiles and pipes illustrates that the method is capable of measuring and inspecting the curvature of surfaces with different levels of precision. Furthermore, it is able to detect physical defects such as flatness, waviness, welds or blobs on the border of items simultaneously, which outweighs previous contributions in defect detection.

## Figures and Tables

**Figure A1.**
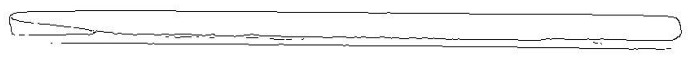
Non-defective ceramic tile after using canny edge detection.

**Figure A2.**
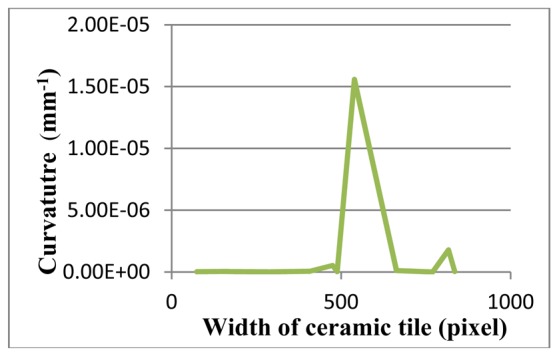
Curvature diagram of a non-defective ceramic tile.

**Figure A3.**
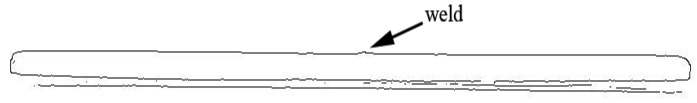
Edge of ceramic tile with a small weld defect in the middle.

**Figure A4.**
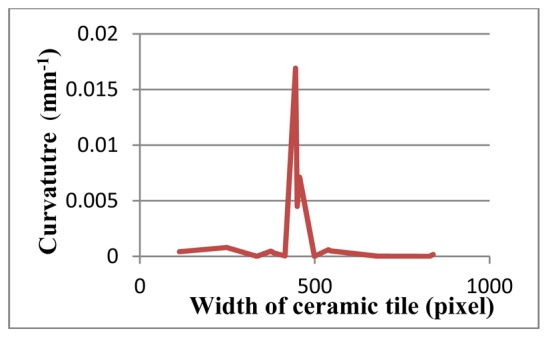
Curvature diagram of a ceramic tile with weld defect on the middle.

**Figure A5.**
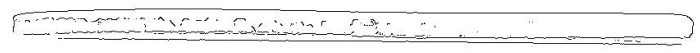
Flatness defected ceramic tile after using edge detection.

**Figure A6.**
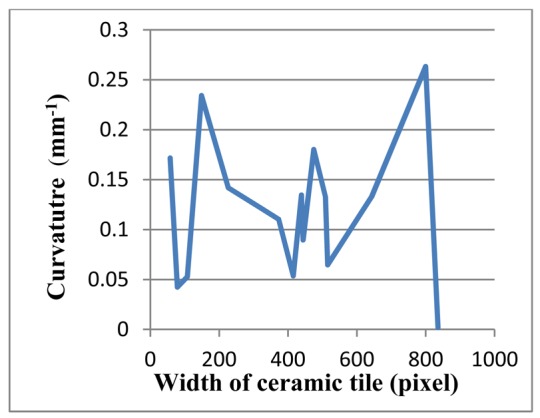
Curvature diagram of flatness defect ceramic tile.

**Figure A7.**
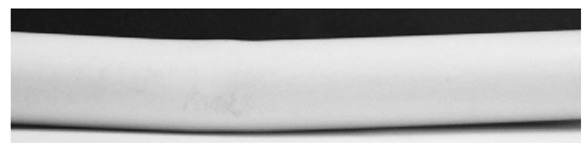
Real image of a pipe showing a curvature defect on the surface.

**Figure A8.**
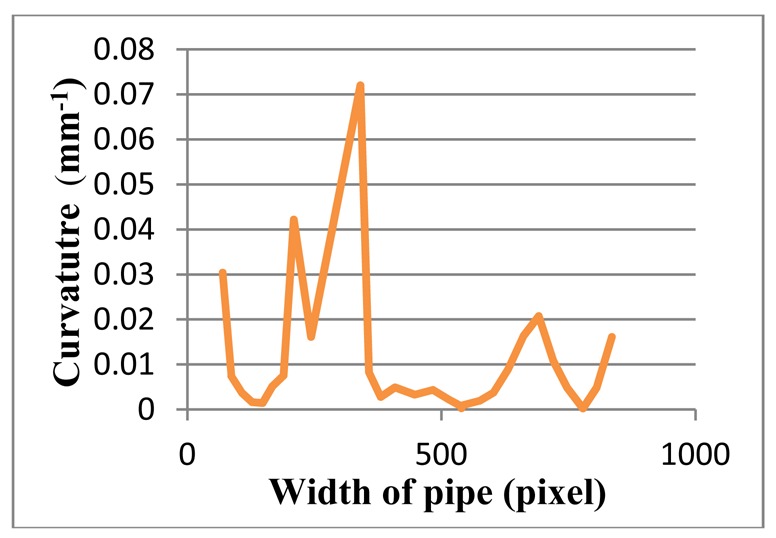
Curvature diagram of a pipe with curvature defect.

**Table 1.** The results of the proposed method for lines and ellipses.

**Shape**	**Max**	**Min**	**Average**	**Standard Deviation**
Line (a)	2.1E-05	8.3E-06	5.4E-06	4.5E-06
Line (b)	6.8E-05	2.7E-08	1.0E-05	1.4E-05
Line (c)	6.9E-04	3.8E-06	1.4E-04	0.00015
Ellipse (a)	0.20264	0.07476	0.04859	0.05732
Ellipse (b)	0.01579	0.00778	0.00702	0.00379

## Appendix

More experimental results of ceramic tiles and pipes with different defects are shown in this section. The results of various defects on different ceramic tiles and pipes show that the proposed method is stable in blob, weld and curvature defects. In contrast, it is unable to detect cracks and scratches on the outer surfaces. In Figure A1, a good ceramic tile with no physical defects on the outer surface after using canny edge detector is shown. The result in Figure A2 illustrates that the curvature of the outer surface is normal and there is no high curvature value in the diagram.

Furthermore, defective ceramic tiles are assessed. For example, Figures A3 and A5 depict ceramic tiles with weld and flatness defects, respectively, and the curvature measurement results in Figures A4 and A6 show that curvatures of ceramic tiles fluctuates around out of the range of valid curvature numbers. Finally, a tested pipe with curvature defect is shown in Figure A7. Besides, the curvature line graph in Figure A8 shows that the curvature of middle of pipe increased by 0.07, showing that this pipe has a curvature defect.
